# Development of a sandwich enzyme-linked immunosorbent assay for dTMP-GH fusion protein by rational immunogen selection

**DOI:** 10.1186/s13568-017-0454-6

**Published:** 2017-07-17

**Authors:** Song Wang, Mingqiang Shen, Shilei Chen, Cheng Wang, Fang Chen, Mo Chen, Gaomei Zhao, Xinze Ran, Tianmin Cheng, Yongping Su, Yang Xu, Junping Wang

**Affiliations:** State Key Laboratory of Trauma, Burns and Combined Injury, Institute of Combined Injury, Chongqing Engineering Research Center for Nanomedicine, College of Preventive Medicine, Third Military Medical University, Chongqing, 400038 People’s Republic of China

**Keywords:** Thrombopoietin mimetic peptide, Human growth hormone, Fusion protein, Sandwich ELISA

## Abstract

dTMP-GH is a chimeric protein containing a tandem dimer of thrombopoietin mimetic peptide (dTMP) fused to human growth hormone (hGH) prepared previously by our team. It shows significant bioactivity in promoting thrombocytopoiesis, but detection of intact dTMP-GH in plasma is still a challenge due to the presence of endogenous hGH. In this study, a rabbit polyclonal antibody with high affinity to dTMP was obtained with a BSA-conjugated immunogen composed of 20 amino acids sequence spanning two TMP and the linker. A monoclonal antibody termed as 3B2 was screened out by using immunizing mice with whole dTMP-GH, which was proved to simultaneously interact with rhGH, TMP-GH, and dTMP-GH, respectively. In this study, we developed a specific and sensitive sandwich enzyme-linked immunosorbent assay (ELISA) with two antibodies (one polyclonal and one HRP-conjugated monoclonal) to quantify dTMP-GH. The polyclonal antibody and HRP-conjugated monoclonal antibody 3B2 were applied as the capture antibody and detection antibody, respectively. A good correlation between ELISA and bicinchoninic acid (BCA) assay in the quantification of diluted dTMP-GH was observed (r^2^ = 0.996). Meanwhile, the standard curve of this ELISA method was found in a linear relationship between 0.2 and 10 ng/mL in the presence of rabbit plasma. In vivo experiments demonstrate that the newly developed method is effective to detect dTMP-GH in rabbits, which paves the way for further pharmacokinetic evaluation.

## Introduction

Thrombopenia has been commonly reported in clinical practice. Thrombopoietin (TPO), a natural ligand for c-Mpl, is the major modulating factor for the formation of megalokaryocyte that plays important roles in the generation of platelets. Nowadays, TPO has been considered as one of the most effective cytokines to raise platelet (Bartley et al. 1994; Kuter et al. 1994; Vainchenker et al. 1998). To date, research and development on recombinant TOP drugs is still in a dilemma due to formation of neutralizing antibodies to the natural TPO (Basser et al. 2002; Li et al. 2001). In 1997, Dower et al. synthesized a TPO-mimetic peptides (TMP) consisting of 14 amino acids that could bind with the c-Mpl with high affinity to promote the proliferation and differentiation of megakaryocytes in vitro (Cwirla et al. 1997). However, because of the small molecular weight, such linear peptides were too short-lived in circulation to be applicated in vivo (Kuter 2007). Recently, several methods have been utilized to extend the half-life of TMP in vivo such as pegylation, as well as binding with Fab or Fc fragments (Broudy and Lin 2004; Frederickson et al. 2006; Kuter 2007). Nevertheless, a time lag of platelet recovery was also observed in the c-Mpl based TMP (Kuter 2002; Kuter and Begley 2002).

Growth hormone (GH), a cytokine contributed to the proliferation and differentiation of various cells, has been reported to play important roles in the platelet generation. In a previous study, significant decrease was detected in the peripheral blood cells (e.g. platelet) in DW/J dwarf mice deficient in GH (Murphy et al. 1992a). Later, high doses of GH was considered to contribute to the hematopoietic function in mice and rats after chemotherapy, radiotherapy and bone marrow transplantation (Carlo-Stella et al. 2004; Chen et al. 2010; Murphy et al. 1992b; Tian et al. 1998; Zhang et al. 2008). Also, recombinant human GH (rhGH) could promote the recovery of platelets after chemotherapy (Sirohi et al. 2007). In our previous study, we designed a tandem dimer of TMP (dTMP) fused to hGH with a purity of up to 98% based on the *Escherichia coli* system by soluble expression (Wang et al. 2013). In addition, the hGH is efficient in promoting the differentiation, especially the terminal differentiation, of human megakaryocytes through stage- and time-specific activation of extracellular signal-regulated kinase (ERK1/2) and protein kinase B (Akt). Moreover, it shows a complementary and synergistic effect with the dTMP on thrombocytopoiesis (Xu et al. 2014).

At present, the detection of hGH fusion protein expression is highly relied on the Western blot assay using anti-hGH antibody. As the anti-hGH antibody could only capture the epitope of the hGH in the fusion protein, it is still a challenge to identify whether the detected protein contains complete N-terminal TMP dimer. To solve this problem, mass spectrum is needed to detect the purified dTMP-GH, but the method is labor-intensive and time-consuming. Moreover, it is not possible to distinguish between the dTMP-GH and natural hGH using anti-hGH antibody. In this study, amino acid sequence corresponding to TMP dimer of dTMP-GH served as immunogen, and a sensitive and specific ELISA method was developed for the quantitative determination of dTMP-GH, which contributed to the measurement of dTMP-GH pharmacokinetics in vivo.

## Materials and methods

### Animals, cells, and regents

Male BALB/c mice (8–12 weeks, weighing 18–22 g) and female New Zealand rabbits were obtained from the animal center of Third Military Medical University. The myeloma cell line SP2/0 cell line was cultured in Dulbecco’s modified Eagle’s medium (Invitrogen, Carlsbad, CA, USA) in a humidified incubator with 5% CO_2_ at 37 °C. The culture media was supplemented with 10% fetal bovine serum (Invitrogen, Carlsbad, CA, USA) and antibiotics (0.1 mg/mL streptomycin and 100 IU/mL penicillin). Immunoaffinity chromatography and protein-A chromatography were purchased from GE healthcare (St. Louis, MO, USA).

### Polypeptide design and conjugation

**Fig. 1:**
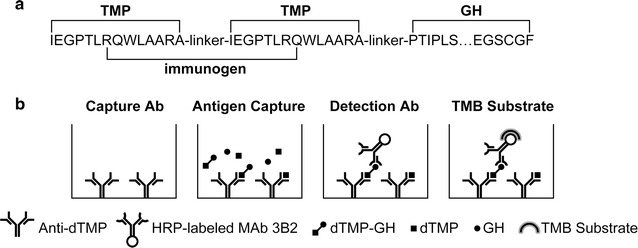
Schematic diagrams of the immunogen amino acids sequence and the ELISA of dTMP-GH detection described in this study. **a** A schematic diagram of the immunogen amino acids sequence. **b** The strategy of the ELISA for dTMP-GH detection

The 20 amino acids sequence spanning two TMP and the linker (N’-**RQWLAARA**-*linker*- IEGPTLRQ-C’) of dTMP-GH served as immunogen (Fig. 1a). The bold part is the amino acids sequence at C-terminal of the first TMP. The normal part is the amino acids sequence at N-terminal of the second TMP. A cysteine residue was added to the C-terminal end of the peptide to facilitate the conjugation to the BSA (Sigma-Aldrich, MO, USA). Immunograde peptide was provided by the GLS Biotech (Shanghai, China). The BSA-conjugated peptide was performed according to the previous study (Hadavi et al. 2010).

### Preparation of monoclonal antibody

The monoclonal antibody was prepared based on standard protocols with slight modifications. Specific pathogen-free BALB/c mice were injected subcutaneously. For the first immunization, 100 μg dTMP-GH or peptide-BSA conjugate dissolved in 100 μL PBS was emulsified with equal volume of complete Freund’s adjuvant (Sigma-Aldrich, St. Louis, MO, USA). Each mice was boosted twice at a 2-week interval with equal amount of immunogen mixed in the same ratio with incomplete Freund’s adjuvant (Sigma-Aldrich, St. Louis, MO, USA). Subsequently, the animals were injected every 3 weeks until reaching the fusion titer, which was identified by indirect ELISA. Three days prior to sacrifice, the animals were injected with 50 μg dTMP-GH dissolved in 50 μL PBS mixed with equal volume of complete Freund’s adjuvant. Mice with the highest titer were sacrificed to obtain the spleens, and then the splenocytes were fused with myeloma cells (SP2/0). The monoclonal antibodies in the supernatant were detected using the indirect ELISA method, with HRG-conjugated goat anti-mouse IgG as secondary antibody. Positive clones were cloned twice by limiting dilution. The ascites from single clones were purified using the protein A chromatography (GE Healthcare, Chalfont, Buckinghamshire, UK).

### Preparation of polyclonal antibody

The polyclonal antibody was prepared according to the general method. Briefly, 300 μg peptide-BSA conjugate dissolved in 1 mL PBS was mixed with equal volume of complete Freund’s adjuvant, and then was injected subcutaneously into the rabbits. Equal amount of peptide-BSA conjugate in incomplete Freund’s complete adjuvant was applied at d4, d29, d43, d57 and d71, respectively. After the 4th immunization (d51), blood (5 mL) of each rabbit was collected from the auricular artery to isolate the serum, and the titer of the polyclonal antibody was determined using the indirect ELISA. After the last immunization, blood (100 mL) was collected from the carotid artery of rabbit, which polyclonal antibody titer meets the requirement to isolate the serum. The polyclonal antibody was purified using the immunoaffinity chromatography and protein-A chromatography. The purity of the antibody was determined using SDS-PAGE.

### Western blot

Western blotting was performed to investigate the reactivity of antibodies obtained. rhGH and dTMP-GH were separated by SDS-PAGE and transferred to the PVDF membrane by a Semi-Dry Transfer System (Bio-Rad, Hercules, CA, USA). The membranes were blocked with 5% non-fat milk at 4° e overnight. Subsequently, the proteins were probed with monoclonal antibodies (1:1000) or polyclonal antibody (1:2000) described previously. Species-specific horseradish peroxidase-conjugated secondary antibodies were used for signal detection with an ECL Plus Kit (Amersham, Pittsburgh, PA, USA).

### Elisa

Anti-dTMP-GH polyclonal antibody dissolved in bicarbonate buffer (50 μM, pH9.6) were added in the 96-well plates (100 μL/well) and incubated at 4 °C overnight. The test sample or purified dTMP-GH diluted by PBS were added (100 μL/well) and incubated at 37 °C for 2 h. After washing with normal saline containing 0.1% Tween 20, the HRG-conjugated monoclonal antibody was added and incubated at 37 °C for 2 h. Afterwards, 100 µL 3, 3′, 5, 5′-tetramethyl benzidine (TMP) was added and incubated at 37 °C for 15 min, followed by terminating the reaction using 50 µL 2M H_2_SO_4_. A wavelength of 450 nm was used for the determination of absorbance.

### Comparison of ELISA and BCA assay

To compare the efficiency of ELISA and BCA assay, purified dTMP-GH sample with a purity of >98% was diluted using distilled water into a concentration of 0.1, 0.25, 0.5, 1, 2.5, 5 and 10 ng/mL, respectively. The standard curves were obtained using the ELISA method or the commercial BCA assay kit, according to the manufacture’s instructions.

### Pharmacokinetics

The dTMP-GH fusion protein diluted with distilled water was administrated into New Zealand rabbit (n = 3) via intramuscular injection (100 μg/kg). The blood was collected from marginal ear vein every half hour after administration. The concentration of dTMP-GH protein was determined using ELISA method. The study protocols were approved by the Ethical Committee of the Third Military Medical University.

## Results

### Generation of efficient monoclonal antibodies

After immunization of BALB/c mice using peptide-BSA, 5 monoclonal antibodies were screened out by indirect ELISA (Table 1). All of these monoclonal antibodies showed cross reactivity to dTMP-GH and Peptide-BSA conjugate. The OD_450_ values of TMP-GH and rhGH wells were similar to BSA, which was used as a negative control. The above data indicated the feasibility of the immunogen. Unfortunately, only 1E10 was IgG. After cloning, only 2D3 was stably established.

**Table 1:** Screening of monoclonal antibodies against peptide-BSA conjugate (OD_450_)

Clone	Type	dTMP-GH	Peptide-BSA conjugate	TMP-GH	rhGH	BSA
1E10	IgG2b	1.857 ± 0.082	2.093 ± 0.094	0.073 ± 0.008	0.099 ± 0.007	0.072 ± 0.005
2C3	IgM	1.918 ± 0.097	1.711 ± 0.065	0.089 ± 0.006	0.091 ± 0.011	0.075 ± 0.005
2D3	IgM	2.166 ± 0.098	2.815 ± 0.085	0.081 ± 0.06	0.084 ± 0.008	0.118 ± 0.007
2E2	IgM	2.101 ± 0.096	1.758 ± 0.077	0.091 ± 0.07	0.082 ± 0.007	0.149 ± 0.009
3G2	IgM	2.245 ± 0.099	1.297 ± 0.067	0.071 ± 0.08	0.075 ± 0.008	0.068 ± 0.007

Values are obtained from three independent experiments conducted in duplicate and shown as the mean ± SD

**Fig. 2:**
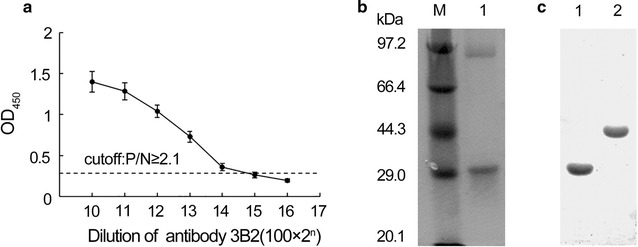
Preparation of anti-dTMP-GH monoclonal antibody 3B2. **a** Titer detection of the monoclonal antibody 3B2. **b** Purity detection of the monoclonal antibody 3B2. *Lane M* protein molecular weight markers; *lane 1* purified monoclonal antibody 3B2. **c** Reactivity of monoclonal antibody 3B2. *Lane 1* dTMP-GH; *lane 2* rhGH

After immunization of BALB/c mice using dTMP-GH, 8 monoclonal antibodies were obtained (Table 2). After cloning, 1A12 and 3B2 with the higher dTMP-GH titer were stably established. In this study, 3B2 with higher titer to TMP-GH and rhGH showing no binding affinity to peptide-BSA conjugate was subjected to purification using protein-A chromatography. Finally, anti-dTMP-GH monoclonal antibody (55.96 mg) with a concentration of 2.798 mg/mL was obtained. The titer was 1:1,600,000 (Fig. 2).

**Table 2:** Screening of monoclonal antibodies against dTMP-GH (OD_450_)

Clone	Type	dTMP-GH	Peptide-BSA conjugate	TMP-GH	rhGH	BSA
1A5	IgG1	2.413 ± 0.102	0.116 ± 0.009	1.670 ± 0.103	0.845 ± 0.085	0.104 ± 0.008
1A12	IgG1	2.046 ± 0.096	0.164 ± 0.010	1.920 ± 0.173	1.042 ± 0.069	0.104 ± 0.008
2B3	IgG1	2.198 ± 0.096	0.079 ± 0.007	1.953 ± 0.122	0.645 ± 0.067	0.097 ± 0.007
2F12	IgG1	2.102 ± 0.089	0.086 ± 0.006	2.638 ± 0.154	0.883 ± 0.076	0.081 ± 0.006
3A8	IgG1	2.017 ± 0.093	0.573 ± 0.028	1.294 ± 0.104	0.909 ± 0.084	0.103 ± 0.008
3B2	IgG1	2.260 ± 0.099	0.064 ± 0.008	2.492 ± 0.153	2.236 ± 0.082	0.090 ± 0.007
3G11	IgG1	2.007 ± 0.084	0.106 ± 0.006	1.932 ± 0.154	1.997 ± 0.096	0.093 ± 0.006
4F11	IgG1	1.935 ± 0.078	0.120 ± 0.012	1.367 ± 0.105	1.050 ± 0.083	0.105 ± 0.008

Values are obtained from three independent experiments conducted in duplicate and shown as the mean ± SD

### Generation of polyclonal antibody

**Fig. 3:**
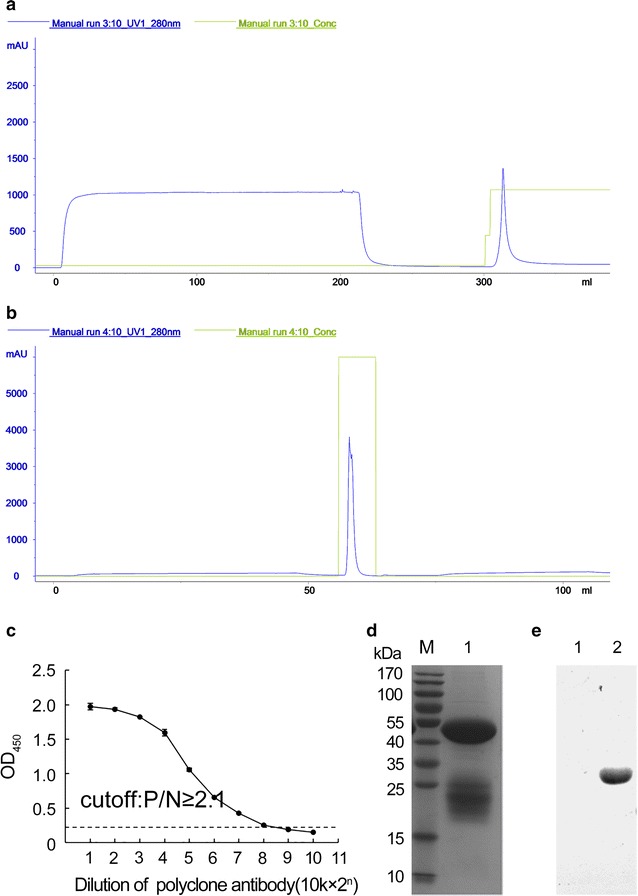
Preparation of polyclonal antibody against peptide-BSA conjugate. **a** Immunoaffinity chromatography purification of the polyclonal antibody. **b** Protein A chromatography purification of the polyclonal antibodies. **c** Titer detection of the polyclonal antibody. **d** Purity detection of the polyclonal antibody. *Lane M* protein molecular weight markers; *lane 1* purified polyclonal antibody. **e** Reactivity of polyclonal antibody against peptide-BSA conjugate. *Lane 1* dTMP-GH; *lane 2* rhGH

After immunization using peptide-BSA conjugate in rabbits, serum was collected and purified. Polyclonal antibody (20.65 mg) with a concentration of 5.90 mg/mL was obtained. The indirect ELISA indicated the titer was up to 1:2,560,000 (Fig. 3).

### Reactivity of the purified antibodies

Western blotting assay was performed to determine the reactivity of the purified antibodies to dTMP-GH and rhGH. The results indicated that 3B2 monoclonal antibody showed reactivity to dTMP-GH and rhGH (Fig. 2c), while anti-dTMP-GH polyclonal antibody could only detect the dTMP-GH fusion protein (Fig. 3e).

### Correlation between of ELISA and BCA assay

**Fig. 4:**
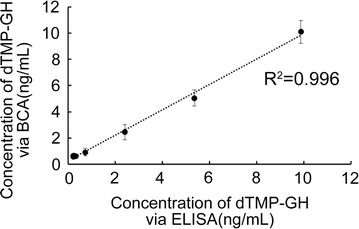
Correlation between ELISA and BCA assays for detection of diluted dTMP-GH

In this part, ELISA and BCA assay was carried out to determine the diluted dTMP-GH, respectively (Fig. 4). The results indicated a satisfactory correlation between ELISA and BCA assay (r^2^ = 0.996).

### Detection of dTMP-GH in rabbit serum

**Fig. 5:**
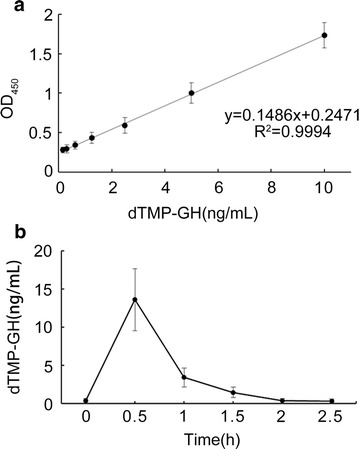
Pharmacokinetics evaluation of dTMP-GH in rabbit. **a** the standard curve of dTMP-GH in rabbit. **b** concentration–time curve of dTMP-GH in rabbit serum after single intramuscular injection (100 μg/kg)

The standard curve of the developed ELISA method in the quantitation of dTMP-GH fusion protein in rabbit serum was in a linear relationship between 0.2 and 10 ng/mL (Fig. 5a). Based on the ELISA method, we determined the level of serum dTMP-GH fusion protein in rabbits, which revealed the maximal concentration was 13.60 ± 4.08 ng/mL 30 min after injection and showed gradual decrease later until reaching the baseline level 2 h after administration (Fig. 5b). This implied that ELISA method was applicable for the pharmacokinetics evaluation of dTMP-GH.

## Discussion

Pharmacokinetic analysis is essential for the research and development of protein therapeutic candidates. Thus, it is indispensable to establish methods for specific candidate detection in vivo. For the development of fusion protein, the binding of the target fragment and some kind of endogenous fragment could contribute to the extension of the circulating half-life and help to attenuate the risks of formation of neutralizing antibodies. Therefore, the specific TMP-based fusion protein could be detected from the expressed or purified product using the commercial antibodies against the part fused with TMP (Fayaz et al. 2016). In our previous study, similar detection had been carried out after the expression and purification of dTMP-GH using anti-hGH antibody (Wang et al. 2013). However, such techniques were not sufficient to some extent. For example, anti-hGH antibody could not testify the dTMP-GH and endogenous hGH. Radioactive isotope has been commonly in the development of TMP-based fusion protein (Broudy and Lin 2004). In our preliminary study, we also detect the pharmacokinetics of dTMP-GH in animals using radioactive isotope (data not shown). However, such method is not only labor-intensive and time-consuming, but also unacceptable in clinical practice on patients. Hall et al. developed ligand-binding mass spectrometry (LBMS) for the detection of TMP fusion protein AMG531 (romiplostim) (Hall et al. 2010) that composed by TMP and the human Fc fragments as a typical “peptibody”, in which those peptibodies were captured by anti-human Fc antibody for further Mass Spectrometry analysis. Such method is effective for the evaluation of AMG531 PKs (Krzyzanski et al. 2013). In order to eliminate the interference of IgG, Hall et al. developed anti-human Fc fragment with no cross reactivity to the human IgG, while intact hGH was integrated in the dTMP-GH, which could not eliminate the effects of endogenous hGH on the metabolites. In addition, MS based method involves additional requirements to the test facility. On this basis, it is urgent to develop a quantitative method for the specific detection of fusion protein in a sensitive manner.

ELISA is effective for the biological assay of protein drugs, but detection focusing on the epitope may not reflect the biological activity region. For dTMP-GH, TMP-dimer is essential for the platelet-forming activity. In order to ensure the developed method could detect the dTMP-GH with complete N-terminal structure, 20 amino acids sequence spanning two TMP and the linker were selected as immunogen, in which C terminus was conjugated with BSA to enhance the immunogenicity of the peptide. However, only one IgM antibody was obtained after immunization of BALB/c mice with peptide-BSA conjugate. Later, the peptide-BSA was used to immunize the rabbits to obtain polyclonal antibody with a titer of 1:2,560,000. Western blotting assay showed such polyclonal antibody could detect the dTMP-GH fusion protein in rabbit serum, with no cross reaction to rhGH and TMP-GH. Thus, using the polyclonal antibody as the capture antibody, we can ensure that the dTMP-GH fusion protein with platelet activating activity is captured, and the interference of endogenous hGH is excluded. Meanwhile, HRP-conjugated monoclonal antibody 3B2 with a titer of 1:1,600,000 served as the coloration antibody, and showed satisfactory reactivity to the TMP-GH and rhGH other than peptide-BSA. This implied that the binding epitope may be localized in hGH. Therefore, application of 3B2 could guarantee the detected protein containing hGH. ELISA method revealed a linear relationship (r^2^ = 0.9994) in a concentration range of 0.2–10 ng/mL. The sensitivity was in line with the requirements of the pharmacokinetic research.

In conclusion, we developed a specific ELISA method using the bioactive region in the dTMP-GH as the immunogen, which could eliminate the interference of endogenous rGH on dTMP-GH detection. In addition, the method could reflect the integrity of dTMP and hGH. All these confirmed the feasibility of ELISA assay in the PKs, which paves the way for the further development of dTMP-GH.

## Abbreviations

TPO: thrombopoietin
TMP: thrombopoietin mimetic peptides
dTMP: dimer of thrombopoietin mimetic peptide
hGH: human growth hormone
ELISA: enzyme-linked immunosorbent assay
BCA: bicinchoninic acid
LBMS: ligand-binding mass spectrometry

## References

[CR1] Bartley TD, Bogenberger J, Hunt P, Li YS, Lu HS, Martin F, Chang MS, Samal B, Nichol JL, Swift S, Johnson MJ, Hsu RY, Parker VP, Suggs S, Skrine JD, Merewether LA, Clogston C, Hsu E, Hokom MM, Hornkohl A, Choi E, Pangelinan M, Sun Y, Mar V, McNinch J, Simonet L, Jacobsen F, Xie C, Shutter J, Chute H, Basu R, Selander L, Trollinger D, Sieu L, Padilla D, Trail G, Elliott G, Izumi R, Covey T, Crouse J, Garcia A, Xu W, Del Castillo J, Biron J, Cole S, Hu MCT, Pacifici R, Ponting I, Saris C, Wen D, Yung YP, Lin H, Rosselman RA (1994). Identification and cloning of a megakaryocyte growth and development factor that is a ligand for the cytokine receptor mpl. Cell.

[CR2] Basser RL, O’Flaherty E, Green M, Edmonds M, Nichol J, Menchaca DM, Cohen B, Begley CG (2002). Development of pancytopenia with neutralizing antibodies to thrombopoietin after multicycle chemotherapy supported by megakaryocyte growth and development factor. Blood.

[CR3] Broudy VC, Lin NL (2004). Amg531 stimulates megakaryopoiesis in vitro by binding to mpl. Cytokine.

[CR4] Carlo-Stella C, Di Nicola M, Milani R, Longoni P, Milanesi M, Bifulco C, Stucchi C, Guidetti A, Cleris L, Formelli F, Garotta G, Gianni AM (2004). Age- and irradiation-associated loss of bone marrow hematopoietic function in mice is reversed by recombinant human growth hormone. Exp Hematol.

[CR5] Chen BJ, Deoliveira D, Spasojevic I, Sempowski GD, Jiang C, Owzar K, Wang X, Gesty-Palmer D, Cline JM, Bourland JD, Dugan G, Meadows SK, Daher P, Muramoto G, Chute JP, Chao NJ (2010). Growth hormone mitigates against lethal irradiation and enhances hematologic and immune recovery in mice and nonhuman primates. PLoS ONE.

[CR6] Cwirla SE, Balasubramanian P, Duffin DJ, Wagstrom CR, Gates CM, Singer SC, Davis AM, Tansik RL, Mattheakis LC, Boytos CM, Schatz PJ, Baccanari DP, Wrighton NC, Barrett RW, Dower WJ (1997). Peptide agonist of the thrombopoietin receptor as potent as the natural cytokine. Science.

[CR7] Fayaz S, Fard-Esfahani P, Golkar M, Allahyari M, Sadeghi S (2016). Expression, purification and biological activity assessment of romiplostim biosimilar peptibody. Daru.

[CR8] Frederickson S, Renshaw MW, Lin B, Smith LM, Calveley P, Springhorn JP, Johnson K, Wang Y, Su X, Shen Y, Bowdish KS (2006). A rationally designed agonist antibody fragment that functionally mimics thrombopoietin. Proc Natl Acad Sci USA.

[CR9] Hadavi R, Zarnani AH, Ahmadvand N, Mahmoudi AR, Bayat AA, Mahmoudian J, Sadeghi MR, Soltanghoraee H, Akhondi MM, Tarahomi M, Jeddi-Tehrani M, Rabbani H (2010). Production of monoclonal antibody against human nestin. Avicenna J Med Biotechnol.

[CR10] Hall MP, Gegg C, Walker K, Spahr C, Ortiz R, Patel V, Yu S, Zhang L, Lu H, DeSilva B, Lee JW (2010). Ligand-binding mass spectrometry to study biotransformation of fusion protein drugs and guide immunoassay development: strategic approach and application to peptibodies targeting the thrombopoietin receptor. AAPS J.

[CR11] Krzyzanski W, Sutjandra L, Perez-Ruixo JJ, Sloey B, Chow AT, Wang YM (2013). Pharmacokinetic and pharmacodynamic modeling of romiplostim in animals. Pharm Res.

[CR12] Kuter DJ (2002). Whatever happened to thrombopoietin?. Transfusion.

[CR13] Kuter DJ (2007). New thrombopoietic growth factors. Blood.

[CR14] Kuter DJ, Begley CG (2002). Recombinant human thrombopoietin: basic biology and evaluation of clinical studies. Blood.

[CR15] Kuter DJ, Beeler DL, Rosenberg RD (1994). The purification of megapoietin: a physiological regulator of megakaryocyte growth and platelet production. Proc Natl Acad Sci USA.

[CR16] Li J, Yang C, Xia Y, Bertino A, Glaspy J, Roberts M, Kuter DJ (2001). Thrombocytopenia caused by the development of antibodies to thrombopoietin. Blood.

[CR17] Murphy WJ, Durum SK, Anver MR, Longo DL (1992). Immunologic and hematologic effects of neuroendocrine hormones. Studies on dw/j dwarf mice. J Immunol.

[CR18] Murphy WJ, Tsarfaty G, Longo DL (1992). Growth hormone exerts hematopoietic growth-promoting effects in vivo and partially counteracts the myelosuppressive effects of azidothymidine. Blood.

[CR19] Sirohi B, Powles R, Morgan G, Treleaven J, Kulkarni S, Horton C, Saso R, Rolfe D, Cook G, Shaw C, Wass J (2007). Use of physiological doses of human growth hormone in haematological patients receiving intensive chemotherapy promotes haematopoietic recovery: a double-blind randomized, placebo-controlled study. Bone Marrow Transpl.

[CR20] Tian ZG, Woody MA, Sun R, Welniak LA, Raziuddin A, Funakoshi S, Tsarfaty G, Longo DL, Murphy WJ (1998). Recombinant human growth hormone promotes hematopoietic reconstitution after syngeneic bone marrow transplantation in mice. Stem Cells.

[CR21] Vainchenker W, Debili N, Norol F, Wendling F (1998). Thrombopoietin and megakaryocyte differentiation. Schweiz Med Wochenschr.

[CR22] Wang S, Shen M, Xu Y, Chen F, Chen M, Chen S, Wang A, Zhang Z, Ran X, Cheng T, Su Y, Wang J (2013). Rational and efficient preparation of a chimeric protein containing a tandem dimer of thrombopoietin mimetic peptide fused to human growth hormone in *Escherichia coli*. Appl Microbiol Biotechnol.

[CR23] Xu Y, Wang S, Shen M, Zhang Z, Chen S, Chen F, Chen M, Zeng D, Wang A, Zhao J, Cheng T, Su Y, Wang J (2014). Hgh promotes megakaryocyte differentiation and exerts a complementary effect with c-mpl ligands on thrombopoiesis. Blood.

[CR24] Zhang Y, Chen J, Liang D, Yuan Y, Wu X (2008). Effects of human growth hormone on haematopoietic recovery of rats receiving chemotherapy. Chemotherapy.

